# Volumetric Stent Expansion Index to Assess Tapering Lesions Using Intravascular Ultrasound and Its Clinical Outcomes

**DOI:** 10.31083/j.rcm2502057

**Published:** 2024-02-05

**Authors:** Xi Wu, Ming-Xing Wu, Hao-Bo Huang, Lei Wang, Zhe Liu, Jie Cai, He Huang

**Affiliations:** ^1^Department of Cardiology, Xiangtan Central Hospital, 411100 Xiangtan, Hunan, China

**Keywords:** intravascular ultrasound, stent expansion, tapered lesion, percutaneous coronary intervention

## Abstract

**Background:**

This study aimed to assess the clinical significance of generating a volumetric stent expansion index for tapering lesions through intravascular ultrasound (IVUS). Previous IVUS studies have used minimal stent area (MSA) to predict adverse outcomes.

**Methods:**

A total of 251 tapering lesions were treated in this study via IVUS guidance in 232 patients. Eight stent expansion indices were evaluated to determine the association of these indices with device-oriented clinical endpoints (DoCEs) after two-year follow-ups. These were the ILUMIEN III and IV standards, the ULTIMATE (Intravascular Ultrasound Guided Drug Eluting Stents Implantation in “All-Comers” Coronary Lesions) standard, the IVUS-XPL (Impact of Intravascular Ultrasound Guidance on the Outcomes of Xience Prime Stents in Long Lesions) standard, the minimal volumetric expansion index (MVEI) using the Huo-Kassab or linear model, the MSA/vessel area at the MSA cross-section, the traditional stent expansion (MSA/mean proximal and distal reference lumen cross-sectional area), and MSA.

**Results:**

The MVEI was the only stent expansion index that correlated significantly with the two-year DoCEs (hazard ratio [HR], 1.91; 95% confidence interval [CI]: 1.16–3.96; *p *= 0.028). In the ROC analysis, the area under the curve for the MVEI was 0.71 (*p* = 0.002), with an optimal cut-off value of 62.2 for predicting the DoCEs.

**Conclusions:**

This is the first study to use IVUS for tapering lesions and demonstrate that the MVEI is an independent predictor of two-year DoCEs.

## 1. Introduction

Coronarytapering lesions (CTLs) refer to a type of lesion where there is a significant mismatch in the lumen diameter between the distal and proximal reference segments of the target lesion [1, 2]. Although interventional and stent techniques have shown rapid progress, the treatment of CTLs remains challenging and is associated with poorer clinical outcomes [3, 4]. The stenting of CTLs is associated with greater in-stent restenosis and risk of stent thrombosis [5]. In light of the adverse events associated with CTLs and the need for more lesion preparation (e.g., using intravascular imaging to assess the vessel size and lesion characteristics) and post-stenting improvement (e.g., using non-compliant balloons with various sizes or pressure) [3], the interventional standard requires urgent modification to improve the outcomes for CTLs.

Extensive research has confirmed the positive effect of stent implantation with guidance from intravascular ultrasound (IVUS) [6, 7]. Adequate stent expansion, measured by IVUS, is recognized as a critical aspect of stent improvement for reducing the failure rate [8]. The minimal stent area (MSA) provides a measure of stent expansion through the use of either optical coherence tomography (OCT) or IVUS. The MSA has been extensively confirmed as a strong predictor of adverse clinical events, with cut-off values for the prediction of stent failure reported as 4.5 to 5.5 ${\mathrm{mm}}^{2}$ [9, 10, 11]. However, regardless of whether OCT or IVUS is used, the value of this traditional methodology is limited if CTLs are not considered. However, the area of under-expansion cannot be accurately assessed. Hence, the volumetric analysis of lumen expansion that considers each CTL is likely to show greater functional precision, and thus, more accurately predict the outcomes [12, 13]. The present study aimed to identify the best stent expansion index (SEI) to evaluate the impact of 2-year percutaneous coronary intervention (PCI) clinical outcomes in coronary tapering lesions.

## 2. Materials and Methods

### 2.1 Study Population

**Fig. 1.**
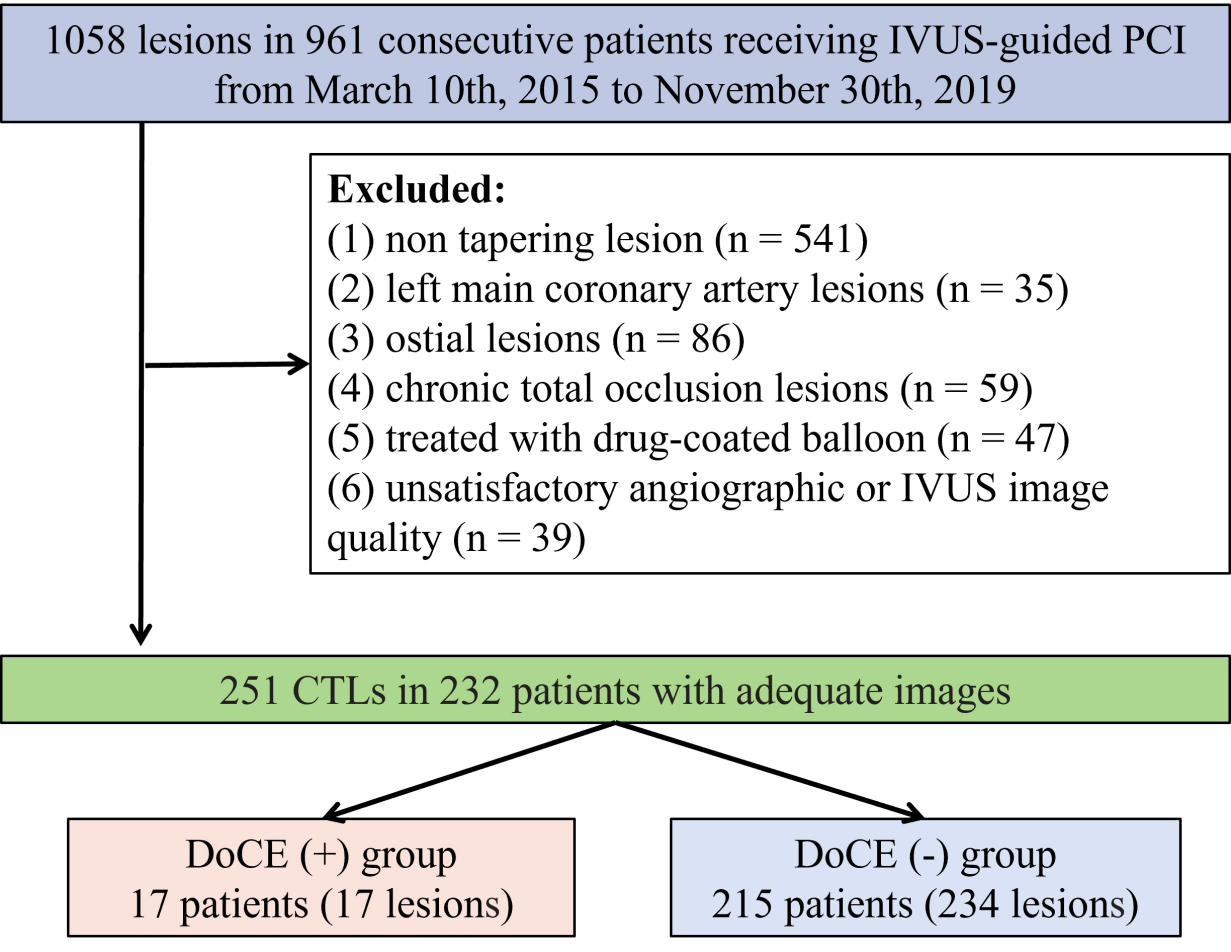
**Schematic of the study flow**. PCI, percutaneous coronary intervention; IVUS, intravascular ultrasound; CTLs, coronary tapering lesions; DoCEs, device-oriented clinical endpoints.

This retrospective observational study was conducted at the Xiangtan Central Hospital from March 2015 to November 2019. A total of 1058 lesions were selected from 961 consecutive patients subjected to IVUS-guided percutaneous coronary intervention (PCI) for *de novo* lesions. Amongst them, 232 cases possessed 251 CTLs. The exclusion criteria were: (1) non-tapering lesions (n = 541), (2) left main coronary artery lesions (n = 35), (3) ostial lesions (n = 86), (4) chronic total occlusion (CTO) lesions (n = 59), (5) administration with drug-coated balloons (n = 47), and (6) non-satisfactory angiographic or IVUS image quality (n = 39) (Fig. 1). CTLs were defined by IVUS and were based on differences in the proximal and distal references for each lesion of ≥1.0 mm, or ≥30% [2]. This study was carried out according to the principles of the Helsinki Declaration and was approved by The Ethical Board of Xiangtan Central Hospital. Written informed consent was obtained from patients prior to the study.

### 2.2 Percutaneous Coronary Intervention

Procedure-associated strategies were decided upon by the operator. Second-generation drug-eluting stents were used in all cases. Preintervention IVUS was employed to analyze CTLs prior to balloon dilatation. Once stenting was complete, another IVUS was carried out to verify the results for stent deployment. For ineffective cases with MSA <4.5 ${\mathrm{mm}}^{2}$, we performed stent improvement using non-compliance balloons and guidance with IVUS and angiography until an acceptable result was achieved, as determined by the final IVUS and angiogram. All patients continued to receive dual antiplatelet therapy (DAPT) for at least 6 months.

### 2.3 Quantitative Coronary Angiography Analysis 

An offline, commercially available software (QAngio® XA, Medis, Leiden, the Netherlands) was employed for quantitative coronary angiography (QCA) of CTLs. QCA analysis included the minimal lumen diameter, percent diameter stenosis, lesion length, reference vessel diameter, calcification, etc. [14]. The three epicardial arteries were divided into left main (LM) (5), distal (3, 4, 8–10, 12, 14, 15), mid (2, 7, 13), and proximal (1, 6, 11) segments, in accordance with the American Heart Association classification [15].

### 2.4 IVUS Image Analysis 

When nitroglycerin (0.1–0.2 mg) was used for intracoronary administration, automated pullback (0.5 mm/s) was employed to obtain the CTL IVUS images (40 MHz OptiCross™, Boston Scientific, Marlborough, MA, USA) for both before and after PCI. Two independent readers who were blinded to patient information evaluated all IVUS images using a frequency domain available offline software (QIvus®, Medis, Leiden, the Netherlands). The CTL IVUS measurements were performed every 1 mm for the administered segment (pre-PCI) and stent, and every 5 mm for the proximal and distal reference segments. We examined the distal and proximal references in the site, reaching the maximal lumen 5 mm distal and proximal to the stented segment. Reference luminal areas were also examined in frames with minimal plaque burden. The calculation for each percent area of stenosis was: ((reference lumen area - minimal lumen area)/reference lumen area) × 100 pre-PCI. The respective volumes were determined in accordance with the Simpson rule [16]. The percentage plaque volume refers to the total plaque/vessel volume for the pre-procedure IVUS investigation. Pre-PCI IVUS qualitative analysis included: superficial calcium (hyperechoic region with acoustic shadow), calcified nodule (protruding and irregular calcium with intimal surface), and attenuated plaque (noncalcified plaque with echo attenuation). Post-PCI IVUS qualitative analysis included: stent edge dissection (intimal, medial, intramural hematoma, or outside the external elastic membrane (EEM)), stent malapposition (blood speckle behind stent struts not overlaying a side branch), and tissue protrusion (plaque and/or thrombi intrusion through the stent struts into the vessel lumen) on post-PCI IVUS [17].

**Fig. 2.**
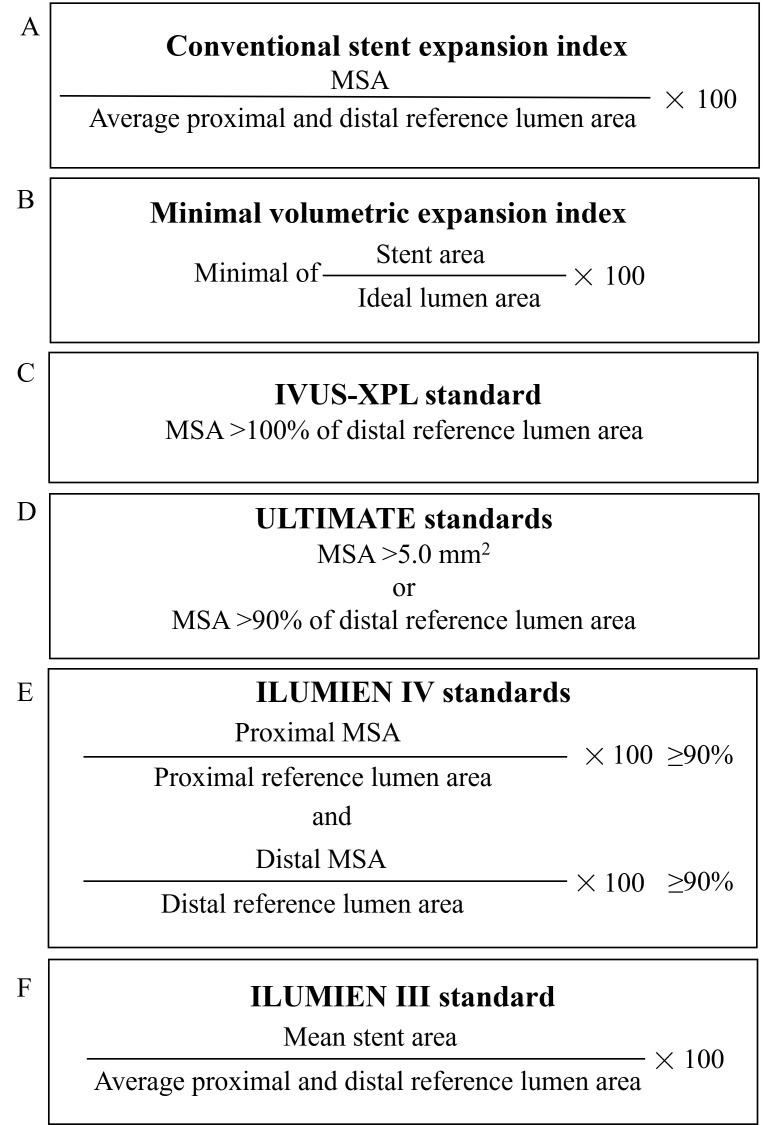
**The calculation formula for stent expansion indices**. MSA, minimal stent area; IVUS-XPL, Impact of Intravascular Ultrasound Guidance on the Outcomes of Xience Prime Stents in Long Lesions; ULTIMATE, Intravascular Ultrasound Guided Drug Eluting Stents Implantation in “All-Comers” Coronary Lesions; ILUMIEN IV, Observational Study of Optical Coherence Tomography in Patients Undergoing Fractional Flow Reserve and Percutaneous Coronary Intervention IV.

The indices for stent expansion were specified in advance and are described below (Fig. 2):

(1) MSA was derived from the automatic minimal cross-sectional lumen area within the post-stented lesion [18].

(2) MSA/vessel area at the MSA cross-sectional [19].

(3) Traditional SEI: MSA/mean proximal and distal reference lumen cross-sectional area.

(4) Minimal volumetric expansion index (MVEI) [13]: (actual lumen area/ideal lumen area × 100) in the minimal value cross-sectional area through the stented site. The ideal lumen cross-sectional area without plaque was calculated using the mathematical relationship for proximal and distal reference cross-sectional areas and side branch diameter (>0.5 mm), as described by Huo *et al*. [20], and referred to as the H–K model. If the vessel has no intermediate side branch (diameter >0.5 mm), the ideal lumen diameter of the uniform tapering vessel was calculated using the linear model [13].

(5) IVUS-XPL standards, calculated by an MSA >100% of the distal reference lumen cross-sectional area [21].

(6) ULTIMATE standards, calculated by an MSA >5.0 ${\mathrm{mm}}^{2}$ or >90% of the distal referencelumen cross-sectional area [7].

(7) ILUMIEN IV standards, calculated by an MSA of the proximal site >90% of the proximal reference lumen cross-sectional area, and an MSA of the distal site >90% of the distal reference lumen cross-sectional area [22].

(8) ILUMIEN III standards, calculated by mean stent expansion: mean stent area (total of stent area/total of stent length)/mean reference lumen cross-sectional area [23].

### 2.5 Clinical Follow-Up

Device-oriented clinical endpoints (DoCEs) included target lesion revascularization (TLR), myocardial infarction (MI) or stent thrombosis associated with the target vessel, and cardiac death [24]. Cardiac death was defined as any death due to cardiac-related causes, procedure-related deaths, and death of unknown cause. MI was reported in accordance with European Society of Cardiology guidelines [25]. TLR refers to an ischemia-driven repeat PCI, or to coronary artery bypass surgery of the target lesion for angiographic target lesion restenosis or ischemia-driven clinical complications. Stent thrombosis refers to either probable or definite stent thrombosis [24]. Periodic clinical follow-up occurred at 6-month intervals through either a telephone interview or a clinical visit. In general, recruited patients were subjected to almost 3-years of clinical follow-ups, and at least one year of follow-ups.

### 2.6 Statistical Analysis

Continuous variables are expressed as mean ± standard deviation when meeting a normal distribution, whereas, for an abnormal distribution, they are described as the median value with the interquartile. Categorical variables are expressed as numbers (percentages). The Mann–Whitney U test or Student’s *t* test was employed for the analysis of continuous outcome data, while the chi-square test or Fisher’s exact test was used for the categorical variables. For lesion-specific variables, continuous and categorical variables were analyzed by generalized estimating equations (GEEs) adopted for clarifying the clustering of multiple lesions in the respective patient. For continuous variables, we employed a GEE model that was subjected to normal distributions, with the expression of the least square means (95% confidence interval). For categorical variables, we employed a GEE model that was subjected to logit link and binomial distributions. Analysis with multivariable marginal Cox proportional hazards was performed using a stepwise selection procedure to identify independent stent expansion indices related to the DoCE. Log-rank and Kaplan–Meier tests were used to compare DoCE incidences between the stent expansion indices. Receiver operating characteristic curve analysis was performed to evaluate the stent expansion indices for their ability to predict DoCEs through the use of minimal under-expansion. We used the Youden index to determine the cut-off value. Associations between CTL morphological parameters, PCI parameters, and stent expansion indicators were analyzed using multivariable linear regression. Statistical significance was defined by two-sided *p* values < 0.05. IBM SPSS Statistics 24.0 (IBM-SPSS Statistics, Chicago, IL, USA) was used to perform statistical analyses.

## 3. Results

### 3.1 Clinical Characteristics and Angiographic and Procedure-Related Findings

A total of 232 consecutive patients with 251 CTLs were assessed. Of these, 17 patients (7.3% of all patients) with 17 lesions (6.8% of all lesions) had 2-year follow-ups for the DoCEs. The average follow-up was 729 days (interquartile range: 705–733 days). As shown in Table 1, no significant differences were observed in any of the clinical characteristics between DoCE(–) and DoCE(+) patient groups. The procedural and angiographic findings were also compared between the two groups. Again, no significant differences were observed between patients who did or did not suffer DoCEs (Table 2).

**Table 1.** **Clinical characteristics**.

Variables		DoCE(+) (n = 17)	DoCE(–) (n = 215)	*p* value
Age, mean ± SD		62.1 ± 9.8	63.4 ± 10.1	0.223
Male, n (%)		5 (29.4)	68 (31.6)	0.461
Body mass index, ${\mathrm{kg/m}}^{2}$, mean ± SD		24.5 ± 3.3	24.8 ± 3.0	0.323
Diabetes mellitus, n (%)		6 (35.3)	63 (29.3)	0.234
Hypertension, n (%)		13 (76.5)	171 (79.5)	0.695
Hyperlipidemia, n (%)		12 (70.6)	145 (67.4)	0.737
Current smoker, n (%)		4 (23.5)	54 (25.1)	0.851
Chronic kidney disease, n (%)		2 (11.8)	23 (10.7)	0.921
Prior PCI, n (%)		1 (5.9)	8 (3.7)	0.693
Prior MI, n (%)		5 (29.4)	70 (32.6)	0.612
Peripheral arterial disease, n (%)		2 (11.8)	21 (9.8)	0.712
Clinical presentation, n (%)				0.804
	STEMI	1 (5.9)	13 (6.0)	
	Non-STEMI	2 (11.8)	27 (12.6)	
	Stable angina	10 (58.8)	124 (57.7)	
	Others	4 (23.5)	51 (23.7)	
Three-vessel coronary disease, n (%)		7 (41.2)	100 (46.5)	0.308
Left ventricular ejection fraction <40%, n (%)		2 (11.8)	31 (14.4)	0.482
Laboratory data				
	Hemoglobin, g/dL, mean ± SD	10.2 ± 1.4	10.1 ± 1.9	0.651
	HbA1c, %, mean ± SD	6.5 ± 1.1	6.4 ± 1.0	0.830
	LDL-C, mg/dL, mean ± SD	108.0 ± 36.1	116.3 ± 42.5	0.439
	HDL-C, mg/dL, mean ± SD	44.1 ± 9.8	45.6 ± 13.1	0.406
	Triglyceride, mg/dL, median (interquartile range)	126.0 (87.1–157.1)	135.0 (88.4–194.1)	0.412
	Creatinine, mg/dL, mean ± SD	0.9 ± 0.3	1.2 ± 1.6	0.225
	eGFR, mL/min/1.73 $m^{2}$, mean ± SD	57.7 ± 18.6	55.2 ± 26.1	0.717
Medication at discharge				
	DAPT, n (%)	17 (100)	215 (100)	1.000
	Beta-blocker, n (%)	11 (64.7)	130 (60.5)	0.721
	ACE inhibitor/ARB, n (%)	10 (58.8)	120 (55.8)	0.801
	Statin, n (%)	16 (94.1)	208 (96.7)	0.887

ACE, angiotensin-converting enzyme; ARB, angiotensin II receptor blocker; DAPT, dual antiplatelet therapy; DoCE, device-oriented clinical endpoint; HDL-C, high density lipoprotein cholesterol; MI, myocardial infarction; LDL-C, low density lipoprotein cholesteroll; PCI, percutaneous coronary intervention; STEMI, ST-segment elevation myocardial infarction; eGFR, estimated glomerular filtration rate; SD, standard deviation; HbA1c, hemoglobin A1c.

**Table 2.** **Baseline angiographic and procedural characteristics**.

Variables		DoCE(+) (n = 17)	DoCE(–) (n = 234)	*p* value
Lesion location, n (%)				0.209
	RCA	2 (11.8)	18 (7.7)	
	LAD	11 (64.7)	160 (68.4)	
	LCx	4 (23.5)	56 (23.9)	
% diameter stenosis, mean ± SD		72.4 ± 15.6	69.7 ± 14.9	0.672
Proximal reference diameter, mm, median (interquartile range)		3.64 (3.36–3.97)	3.78 (3.40–3.99)	0.174
Distal reference diameter, mm, median (interquartile range)		2.38 (2.06–2.69)	2.45 (2.15–2.81)	0.136
Stent diameter, mm, mean ± SD		3.5 ± 0.6	3.3 ± 0.5	0.114
Stent length, mm, mean ± SD		29.4 ± 6.4	29.7 ± 7.3	0.854
Multiple stents, n (%)		13 (76.5)	175 (74.8)	0.628
Predilatation, n (%)		11 (64.7)	156 (66.7)	0.271
Postdilatation, n (%)		17 (100)	234 (100)	1.000
Maximal inflation pressure, atm, mean ± SD		18.5 ± 2.5	18.1 ± 1.9	0.708

LAD, left anterior descending artery; LCx, left circumflex artery; RCA, right coronary artery; DoCE, device-oriented clinical endpoint; SD, standard deviation.

### 3.2 Associations between Stent Expansion Indices and DoCE

**Fig. 3.**
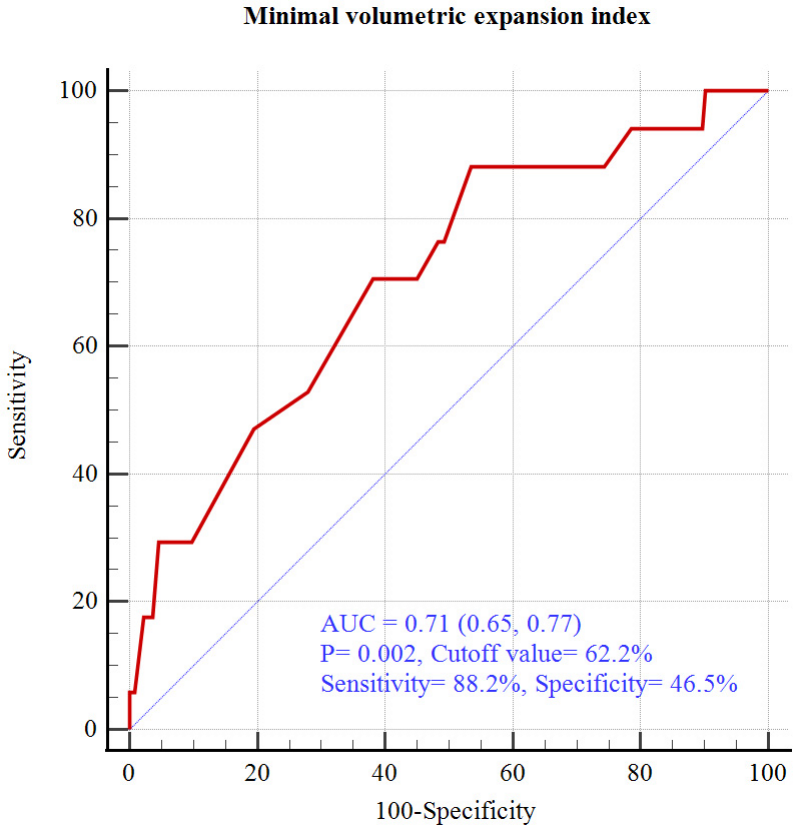
**Receiver operating characteristic curve analysis**. AUC, area under curve.

**Fig. 4.**
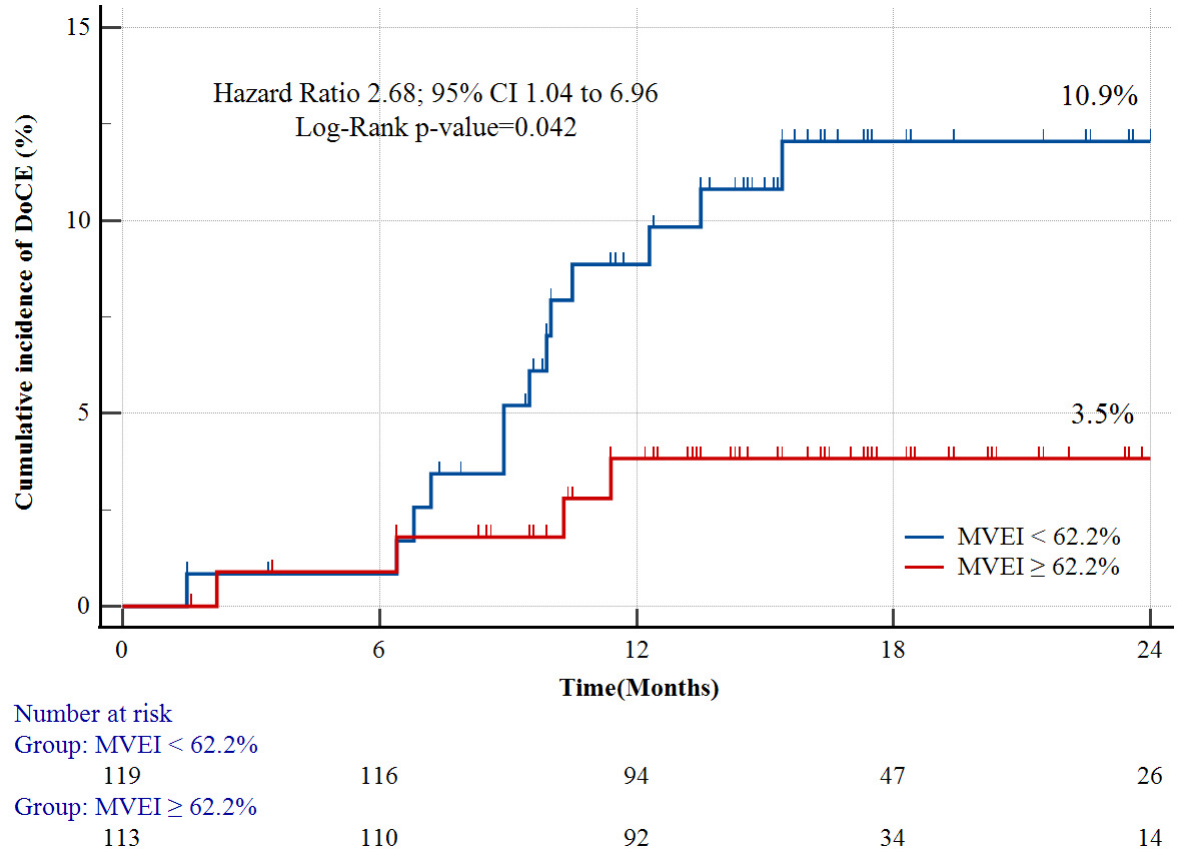
**Two-year Kaplan–Meier curves for DoCEs**. DoCEs, device-oriented clinical endpoints; CI, confidence interval; MVEI, minimal volumetric expansion index.

**Fig. 5.**
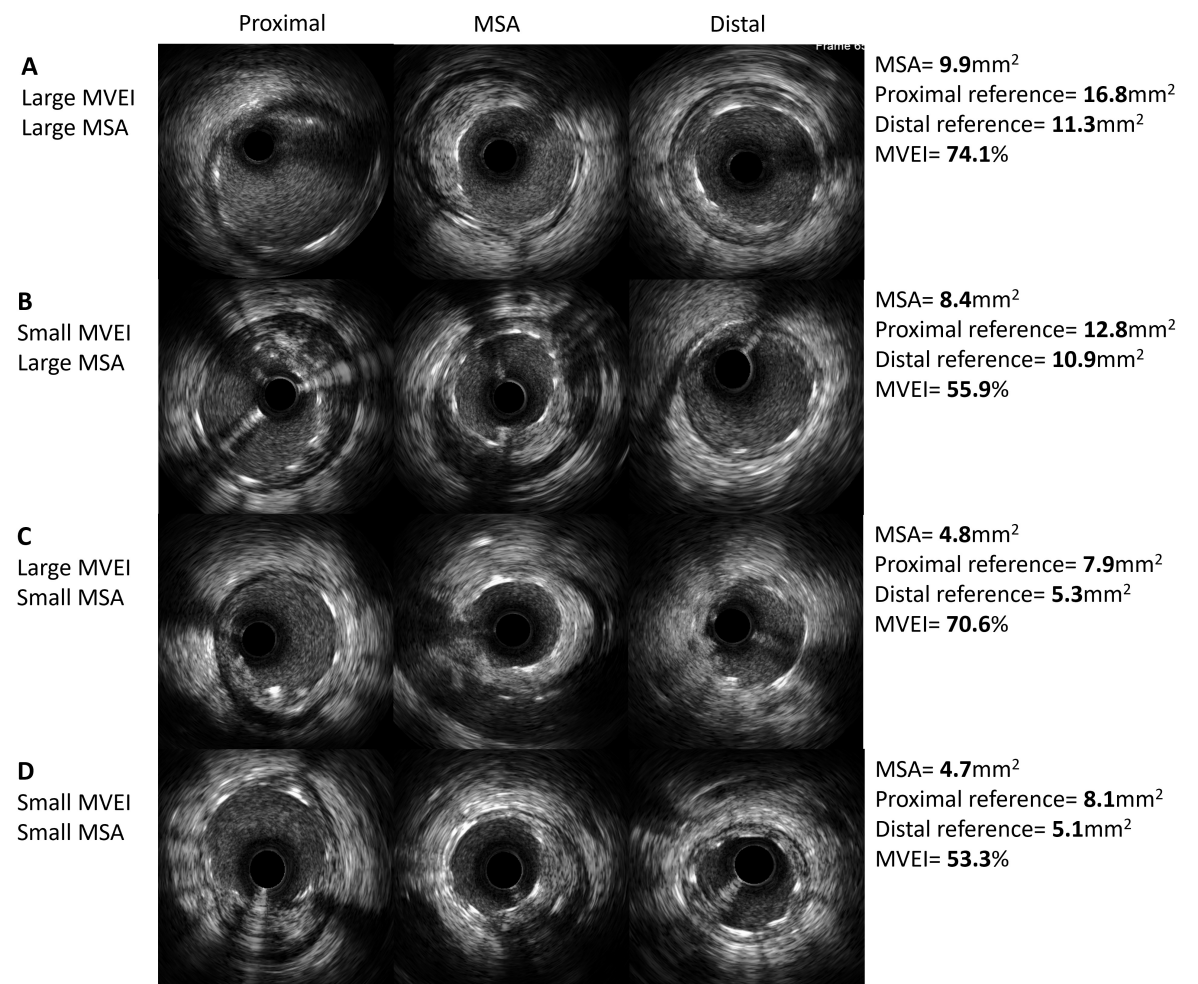
**Representative IVUS images with different patterns of MVEI**. (A) Both MSA and MVEI are large. (B) MSA is large and MVEI is small. (C) MSA is small and MVEI is large. (D) Both MSA and MVEI are small. MVEI, minimal volumetric expansion index; IVUS, intravascular ultrasound; MSA, minimal stent area.

The final overall IVUS MSA after PCI was examined as 5.9 ± 1.6 ${\mathrm{mm}}^{2}$. Table 3 shows the results for lesions with IVUS prior to PCI. There were no significant differences in lesions at the MSA site between patients with or without DoCEs. However, the MVEI calculated by the linear model or the H–K model had significantly fewer lesions in the DoCE(+) patients compared to the DoCE(–) patients. Among the different stent expansion indices, only MVEI (hazard ratio [HR], 1.91; 95% CI 1.16–3.96; *p* = 0.028) was significantly associated with an increased risk of DoCEs in the multivariable analysis (Table 4). Higher balloon inflation pressures were negatively related to the volumetric expansion index (HR: 0.28; 95% CI: 0.10–0.47; *p* = 0.01) (Table 5). Receiver operating characteristic analysis revealed that the optimal MVEI cut-off value for predicting a DoCE was 62.2% (area under the curve [AUC]: 0.71; 95% CI: 0.65–0.77) (Fig. 3). The Kaplan–Meier analysis of the DoCE after the two-year follow-up, in relation to the MVEI is shown in Fig. 4. A significant difference was observed in the incidence rate of 2-year DoCEs between patients with MVEI <62.2% and MVEI ≥62.2% (10.9% *vs* 3.5%; *p* < 0.011), as shown in Table 6. Summaries of representative cases for MVEI are shown in Fig. 5.

**Table 3.** **Intravascular ultrasound findings**.

Variables			DoCE(+) (n = 17)	DoCE(–) (n = 234)	*p* value
Pre-PCI IVUS					
	Minimal luminal area site analysis				
		Luminal area, ${\mathrm{mm}}^{2}$, median (interquartile range)	2.8 (2.6–3.0)	2.7 (2.5–2.9)	0.295
		Vessel area, ${\mathrm{mm}}^{2}$, median (interquartile range)	13.9 (11.8–15.7)	13.6 (13.2–14.3)	0.783
		Plaque burden, %, median (interquartile range)	77.1 (72.4–82.1)	76.6 (73.2–79.7)	0.743
	Volumetric analysis				
		Mean luminal area, ${\mathrm{mm}}^{3}$/mm, median (interquartile range)	5.7 (5.4–5.9)	5.6 (5.2–6.0)	0.383
		Mean vessel area, ${\mathrm{mm}}^{3}$/mm, median (interquartile range)	13.9 (12.4–15.5)	14.1 (13.4–14.9)	0.642
		Plaque volume, %, median (interquartile range)	62.1 (59.2–64.9)	61.2 (59.1–63.5)	0.211
		Mean reference area, ${\mathrm{mm}}^{2}$, mean ± SD	6.13 ± 2.23	6.37 ± 2.42	0.374
		Mean distal reference area, ${\mathrm{mm}}^{2}$, mean ± SD	4.08 ± 2.33	5.22 ± 2.39	0.131
		Mean proximal reference area, ${\mathrm{mm}}^{2}$, mean ± SD	6.93 ± 2.64	7.63 ± 3.23	0.318
		Superficial calcium, n (%)	2 (11.8)	24 (10.3)	0.712
		Calcified nodule, n (%)	1 (5.9)	14 (6.0)	0.832
		Attenuated plaque, n (%)	4 (23.5)	47 (20.1)	0.214
Post-PCI IVUS					
		Minimal stent area, ${\mathrm{mm}}^{2}$, mean ± SD	5.8 ± 1.4	6.0 ± 1.8	0.327
		MSA/vessel area at the MSA, %, median (interquartile range)	47.9 (39.3–54.5)	50.1 (44.1–55.9)	0.072
		Conventional stent expansion, %, median (interquartile range)	75.7 (72.4–78.9)	74.6 (72.8–76.1)	0.793
		Minimal volumetric expansion index, %, median (interquartile range)	65.3 (59.7–70.9)	72.1 (67.2–76.3)	0.001
		IVUS-XPL criteria, n (%)	4 (23.5)	47 (20.0)	0.643
		ULTIMATE criteria, n (%)	5 (29.4)	68 (29.0)	0.982
		ILUMIEN IV criteria, n (%)	2 (11.8)	23 (9.8)	0.492
		ILUMIEN III criteria, %, mean ± SD	103.5 ± 16.3	97.2 ± 15.6	0.314
		Tissue protrusion, n (%)	6 (35.3)	79 (33.7)	0.519
		Stent edge dissection, n (%)	3 (17.6)	36 (15.4)	0.322
		Acute stent malapposition, n (%)	0 (0)	2 (0.09)	0.737

IVUS, intravascular ultrasound; IVUS-XPL, Impact of Intravascular Ultrasound Guidance on the Outcomes of Xience Prime Stents in Long Lesions; MSA, minimal stent area; ULTIMATE, Intravascular Ultrasound Guided Drug Eluting Stents Implantation in “All-Comers” Coronary Lesions; DoCE, device-oriented clinical endpoint; PCI, percutaneous coronary intervention; SD, standard deviation; ILUMIEN, Observational Study of Optical Coherence Tomography in Patients Undergoing Fractional Flow Reserve and Percutaneous Coronary Intervention.

**Table 4.** **Association between SEI and DoCE in multivariable Cox proportional hazards model**.

Variables	Hazard ratio	95% Confidence interval	*p* value
Minimal stent area, ${\mathrm{mm}}^{2}$	0.95	0.89–1.12	0.655
MSA/vessel area at the MSA, per 10%	0.78	0.62–1.32	0.314
Conventional stent expansion, %	1.04	0.88–1.32	0.745
Minimal volumetric expansion index, per 10%	1.91	1.16–3.96	0.028
IVUS-XPL criteria	1.61	0.74–3.35	0.178
ULTIMATE criteria	0.92	0.53–1.78	0.793
ILUMIEN IV criteria	0.74	0.24–2.45	0.688
ILUMIEN III criteria, per 10%	1.53	0.35–7.34	0.653

SEI, stent expansion index; DoCE, device-oriented clinical endpoint; IVUS, intravascular ultrasound; IVUS-XPL, Impact of Intravascular Ultrasound Guidance on the Outcomes of Xience Prime Stents in Long Lesions; MSA, minimal stent area; ULTIMATE, Intravascular Ultrasound Guided Drug Eluting Stents Implantation in “All-Comers” Coronary Lesions; ILUMIEN IV, Observational Study of Optical Coherence Tomography in Patients Undergoing Fractional Flow Reserve and Percutaneous Coronary Intervention IV.

**Table 5.** **Relationship between minimal volumetric expansion index, angiographic, and IVUS findings using multivariable logistic regression**.

Variables	Hazard ratio	95% Confidence interval	*p* value
Maximal inflation pressure, atm	0.28	0.10–0.47	0.001
Multiple stents	1.57	0.78–3.32	0.682
Plaque volume, per 10%	1.29	0.77–2.92	0.326
Lesion length, per 10 mm	1.08	0.51–2.59	0.474

IVUS, intravascular ultrasound.

**Table 6.** **DoCEs between minimal volumetric expansion indices <62.2% and ≥62.2%**.

Variables	MVEI <62.2%	MVEI ≥62.2%	*p* value
Patients level	(n = 119)	(n = 113)	
DoCEs, n (%)	13 (10.9)	4 (3.5)	0.011
Cardiac death, n (%)	0 (0)	0 (0)	–
Target vessel-related myocardial infarction, n (%)	3 (2.5)	2 (1.8)	0.702
Stent thrombosis, n (%)	2 (1.7)	1 (0.9)	0.649
Target lesion revascularization, n (%)	8 (6.7)	1 (0.9)	0.021
Lesions level	(n = 126)	(n = 125)	
DoCEs, n (%)	13 (10.3)	4 (3.2)	0.018
Cardiac death, n (%)	0 (0)	0 (0)	–
Target vessel-related myocardial infarction, n (%)	3 (2.4)	2 (1.6)	0.820
Stent thrombosis, n (%)	2 (1.6)	1 (0.8)	0.862
Target lesion revascularization, n (%)	8 (6.3)	1 (0.8)	0.038

DoCEs, device-oriented clinical endpoints; MVEI, minimal volumetric expansion index.

## 4. Discussion

This is the first evaluation of stent expansions in CTLs. We report an algorithm for volumetric analysis, which is based on IVUS investigations that assess stent expansions. The major findings of this study were firstly that DoCE(+) patients showed lower MVEIs compared to DoCE(–) patients. Secondly, MVEI was found to be the only independent determinant of DoCEs, with none of the other stent expansion indices showing a significant association with clinical outcomes. Thirdly, higher balloon inflation pressures correlated with larger MVEI ratios. Finally, the optimal MVEI cut-off value for predicting DoCEs was 62.2%.

### 4.1 Treatment of CTLs

CTLs with reference lumen cross-sectional area mismatching remain difficult to treat by interventional cardiologists, with no optimal interventional strategy confirmed as yet. The remodeling of vessels at the reference site of the target lesion is the primary treatment for CTLs [26]. Although self-expandable stents and tapered stents are employed to revascularize CTLs [27, 28], they have yet to be extensively employed. Moreover, in contrast to existing balloons and symmetrical stents, their efficacy has also yet to be confirmed. Due to the effect of the symmetrical design, and without considering strategies involving stents and balloons, PCI, in terms of tapering lesions through the application of self-expandable stents and tapered stents that are symmetrical, is subject to dissection and overstretching risks within the distal segment, or to incomplete stent apposition and thrombus formation at proximal sites of the tapering lesion. The above sub-optimal conditions for tapering lesions are likely to trigger common PCI complications, such as in-stent restenosis and stent thrombosis [3]. The present study highlights the use of the volumetric expansion index under the guidance of IVUS to assess whether tapering stent expansion is important for reducing adverse clinical outcomes. In addition, the volumetric analysis algorithm could also optimize the success of stent implantations with symmetrical devices.

### 4.2 Absolute Stent Expansion and Clinical Outcomes

Current research suggests that PCI following IVUS guidance can optimize stent expansion by providing accurate lesion assessment, pre-stenting preparation, and post-dilation improvement. MSA is capable of estimating absolute stent expansion and is known to be a critical predictor of future stent failures in IVUS and OCT research. The optimal MSA cut-off values for the prediction of adverse clinical outcomes are reported to be 4.5 to 5.5 ${\mathrm{mm}}^{2}$ [10, 29, 30, 31]. Data from a randomized trial with 804 patients who received a long (≥28 mm length) drug-eluting stent implant used IVUS to identify an MSA of <5.0 ${\mathrm{mm}}^{2}$ as a threshold for predicting future clinical outcomes [9]. However, MSA is mainly determined by the reference cross-sectional lumen diameter of the target lesion, although this varies depending on the distribution of the three main epicardial coronary arteries and lesions. The concept of “bigger is better” does not apply to all lesions. For example, a lesion at the distal end of the right coronary artery (e.g., 2.5 mm diameter) cannot easily achieve an MSA >5.0 ${\mathrm{mm}}^{2}$ after a stent is implanted and with optimized post-dilation. The final overall MSA here was 5.9 ± 1.6 ${\mathrm{mm}}^{2}$, thereby suggesting a favorable IVUS-guided stent expansion. This is likely the reason why MSA was unable to predict further stent failures in the current cohort. The above result demonstrates that the independent use of MSA has a limited application for individual cases.

### 4.3 Relative Expansion of Stents and Clinical Outcomes

A uniform standard for comparing the minimal luminal area in the intravascular imaging-guided stent has yet to be established for the proximal or distal reference luminal area, or the mean luminal area. Meneveau *et al*. [31], reported that an optimal cut-off value for stent expansion >79.4% and a minimal luminal area >5.44 ${\mathrm{mm}}^{2}$ could predict a final fractional flow reserve (FFR) >0.90. In addition, the recent expert consensus document [8] recommended a value >80% to improve clinical outcomes, in terms of the MSA/mean reference luminal area. However, for small vessels, an MSA/mean reference luminal area of >80% is not feasible [32]. Furthermore, pooled data from the ADAPTDES (dual antiplatelet therapy evaluation that involves drug-eluting stents) study found that neither MSA nor traditional expansion indices affected the two-year DoCEs [19]. Instead, the IVUS-directed MSA/vessel area ratio at the MSA section was significantly associated with adverse two-year clinical outcomes when the value was <38.9%. Nakamura *et al*. [13] investigated the relationship between the incidence of DoCEs at 1-year and the H–K model-derived minimal index for volumetric stent expansion and post-stent FFR. Consistent with the present study, the authors reported that a volumetric analysis model that considers vessel tapering is a better predictor of final FFR and clinical events. In contrast, a different cut-off value was reported for OCT-derived volumetric parameters (74.0% in Katsura *et al*. [12] and 62.2% in the present cohort). One explanation may be that the rate of post-dilatation in the relevant segment of the stent was higher in our study than in Katsura *et al*. [12] (100% *vs*. 80.5% ). This may reduce the frequency of stents under expansion and increase the expansion volume of the stents. In summary, the current research indicates that vessel tapering or vessel remodeling in the volumetric stent expansion index is an important criterion for post-stent improvement and for the prediction of adverse clinical events.

### 4.4 Associations between Post-Stent Dilatation and Clinical Outcomes

The choice of a symmetrical stent for achieving the favorable conformation of a tapering lesion is very challenging in real-world practice [3]. The use of post-stenting improvements with a larger-sized balloon or a greater pressure of inflation helps to address tapering lesions but causes higher rates of stent failure [33, 34, 35]. The primary clinical endpoint was higher in the present study (7.3% of patients) than in previous clinical PCI studies (2.9%–3.9% [13, 19, 36]). All cases in our cohort were accepted post-dilatation and with a higher in-stent balloon inflation pressure (average >18 atm), which reduced both the stent under-expansion and the severe incomplete stent apposition. An animal study [37] reported that adventitial myofibroblasts are a vital feature of atherosclerosis in coronary arteries. Following damage to the vessel wall, proliferating cells synthesize growth factors and migrate into the vascular intima. A serial IVUS observational study [38] found that vascular morphology and vascular stretching are altered after stent implantation. The total vascular area post-PCI was correlated with in-stent neointimal proliferation rather than with the lumen or plaque area. Therefore, a stretch or injury to the adventitia rather than the intimal section is important for neointimal growth. Appropriate stretching of the total vascular area plays a significant role in preventing late in-stent neointimal hyperplastic growth. The incidence of adverse clinical events was higher in our study, suggesting that excessive in-stent expansion may lead to vascular injury and to an increased risk of adverse events. Furthermore, the minimal total vascular expansion area should be achieved through post-dilatation.

## 5. Limitations

Firstly, this was a non-randomized observational investigation that was conducted at a single center. No independent third party was employed to assess the incidence of adverse clinical events. Secondly, some potential selection bias may have occurred in the present study due to the presence of insufficient IVUS images. Thirdly, further additional investigations are required to determine the clinical outcomes after using larger balloons and/or higher pressures in the tapering lesions. Fourth, the measurement of the side branch luminal diameter may be affected by guidewire bias in tortuous vessels and by the oblique orientation of the IVUS catheter. Finally, only 17 patients had a DoCE in the present study. Hence, the effect of the MVIE on clinical events cannot be determined.

## 6. Conclusions

For CTLs, MVEI was superior at predicting 2-year DoCEs compared to the traditional methodologies of stent expansion.

## Data Availability

The datasets generated during and/or analyzed during the current study are available from the corresponding author on reasonable request.
